# Antimicrobial Unusual Small Molecules from Marine *Streptomyces* spp.

**DOI:** 10.3390/ijms26167771

**Published:** 2025-08-12

**Authors:** M. A. Mojid Mondol, Tanvir Islam Shovo, Abul Hasnat Md. Shamim, Abdullah Al Azam

**Affiliations:** 1 Food, Nutrition and Natural Product Chemistry Laboratory, School of Science and Technology, Bangladesh Open University, Gazipur 1705, Bangladesh; tanvirshovo.ts@gmail.com (T.I.S.); abdullahazam1856@gmail.com (A.A.A.); 2 Agriculture Research Laboratory, School of Agriculture and Rural Development, Bangladesh Open University, Gazipur 1705, Bangladesh; shamim@bou.ac.bd

**Keywords:** marine natural products, marine microorganisms, structure elucidation, antimicrobial activity

## Abstract

The widespread emergence of resistant pathogenic microorganisms are diminishing the effectiveness of existing antimicrobial drugs, posing an enormous threat to global public health. This phenomenon, known as antimicrobial resistance (AMR), is primarily driven by the misuse and overuse of antimicrobial drugs. Natural product researchers around the globe, in response to antibiotics resistance, are searching for new antimicrobial lead compounds from unexplored or underexplored ecological niches such as the marine environment. In order to isolate new antimicrobial lead compounds, two *Streptomyces* spp. were isolated from marine sediment samples by a serial dilution technique and subsequently cultured in modified Bennett’s broth medium. Repeated chromatographic steps of ethyl acetate (EtOAc) extracts obtained from the culture broth led to the isolation of a new compound with an unusual chemical skeleton, streptopiperithiazol (**1**), and a synthetically known (**2**) compound. These compounds were characterized by the extensive analysis of 1D and 2D spectroscopic as well as HRMS data. The absolute configuration of **1** was unresolved due to limited yield and lack of proper facilities for taking CD and ECD spectra. In vitro activity study of compounds **1** and **2** revealed that these compounds had better activity against Gram-positive bacteria than Gram-negative bacteria and yeast.

## 1. Introduction

In 1929, the history of antibiotics began with the discovery of the first antibiotic, penicillin, by Alexander Fleming; it is a type of drug that destroys or limits the growth of harmful bacteria [1]. After discovery, antibiotics were indeed hailed as “magic bullets” because of their ability to cure bacterial infections without harming the host and were considered a remarkable medical discovery of the 20th century [2]. For more than one decade after its discovery, antibiotics remained unbeaten in controlling diseases caused by pathogenic microorganisms [3,4]. After the discovery of most antimicrobial drugs (1940s to 1960s), the scientists assumed that these antimicrobials were sufficient to treat infectious diseases of human beings. This concept has been proved to be wrong due to emerging multidrug-resistant pathogens that are resistant to the existing antimicrobial agents [5].

While antibiotics were highly effective for decades in treating infectious diseases, their effectiveness has diminished due to the emergence of multidrug-resistant pathogens, primarily driven by underuse, overuse, and misuse of antimicrobial drugs in humans, animals, and plants [6]. Gram-negative pathogenic microorganisms are particularly worrisome because they are gaining resistance to nearly all the antibiotic drugs available in practice, creating situations reminiscent of the pre-antibiotic era [7,8,9]. More than 70% of all pathogens are thought to be resistant to at least one commercially available antibiotic [10,11]. The emergence of multidrug-resistant pathogens has made many previously treatable infections difficult or impossible to manage. The rise of pathogenic microorganisms resistant to antibiotics poses an enormous threat to global health (affecting all countries), leading to increased mortality, disability, and treatment costs.

Natural products and their semisynthetic derivatives have played a vital role in developing antimicrobial drugs, especially in the last 20 years [12], where diverse sources, including plants, fungi, and bacteria, presented more than 80% of reported naturally derived antibiotics. These antibiotics were found to be active in controlling pathogens through different mechanisms [13]. The discovery of new antimicrobial drugs from natural sources has declined significantly due to the frequent rediscovery of already known natural products. This issue is compounded by the rise of antimicrobial resistance and the lack of new antibiotic development to address the drug resistance problem. So, discovering new sources of bioactive natural products is crucial for developing novel antimicrobial drugs that can combat drug-resistant pathogens and act through different mechanisms.

Natural products have been used for the treatment of human ailments since the beginning of mankind. The ocean remains as one such treasure for natural products. Although more than 100 drugs exist today that come from terrestrial microorganisms, arguably the most important drug in medicine began to dwindle nearly 10 years ago due to repeated isolation of known natural products from terrestrial plants and microbial sources [14]. The oceans cover approximately 70% of Earth’s surface and contain about 97% of its water. The ocean is indeed home to the most diverse range of living organisms on Earth, with an estimated 80% of global biodiversity residing within its waters. The depth of the ocean is up to 11,000 m, providing a vast and varied range of habitats for different species. 

Marine organisms thrive in harsh conditions, such as high salt concentrations, high pressure, high pH, and nutrition depletion, as well as varying temperature and light intensity in the marine environment compared to their terrestrial counter parts. Marine organisms have evolved with unique and diverse adaptation capabilities driven by harsh underwater living conditions. These adaptations include unique metabolic pathways, sensory and defense mechanisms, reproductive systems, and physiological processes [15].

Marine microorganisms produce a wide array of bioactive compounds that serve various purposes, including killing predators, competing with other organisms for resources like nutrients and space, and affecting sensory perception [16]. These compounds, often called secondary metabolites, are not essential for the microorganism’s basic survival but provide a competitive advantage in their complex marine environment. Marine organisms, particularly microorganisms, produce a wide array of unique bioactive compounds, including secondary metabolites, that are not found in terrestrial species [17]. These compounds often have high biological and structural activity, making them valuable for drug discovery and other applications [18].

**Figure 1:**
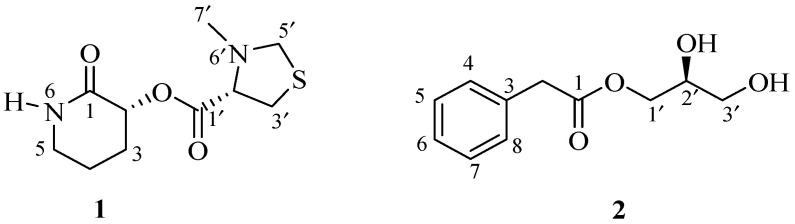
Chemical structures of compounds **1** and **2**.

The need for new lead structures has stimulated the exploration of marine organisms for bioactive secondary metabolites. Antibiotic-producing marine bacteria are found in many taxonomic groups, with the majority of antibiotics originating from actinomycetes, particularly *Streptomyces* sp. [19]. Marine microorganisms are considered a rich source of unique chemical scaffolds; they produce bioactive compounds with structural frameworks not typically found in terrestrial sources [20]. Here, we report the isolation, structure determination and antimicrobial activity of one unusual new (**1**) and one synthetically known (**2**) small molecules (Figure 1) isolated from the culture broths of two marine *Streptomyces* spp.

## 2. Results and Discussion

Both the strains 04DH31 and 06CH80 were white in color and showed aerial mycelium, substrate mycelium, mycelial fragmentation, and spore chain morphology under a light microscope (1000×). Strain 04DH31 was grown in a round shape but was void in the middle just like a donut, whereas the 06CH80 strain showed a long aerial mycelium that was filamentous and rectiflexible in the arrangement.

The positive-ion mode HRESIMS data of streptopiperithiazol (**1**) showed a pseudo-molecular ion [M + Na]^+^ peak at *m*/*z* 267.0777 (Supporting Information), consistent with the molecular formula C_10_H_16_N_2_O_3_S, requiring four degrees of unsaturation. An absorption maxima observed in the UV spectrum at λ_max_ 202 nm indicated the presence of carbonyl (C=O) chromophore. This was also supported by the absorption band for the carbonyl (1652 cm^−1^) group in the IR spectrum. The ^13^C NMR spectrum revealed 10 signals, which were ascribed to two carbonyl carbons, two methine carbons, five methylene carbons, and one methyl carbon with the help of an HSQC spectrum (Table 1). The extensive analysis of 1D and 2D NMR of 1 revealed two structural units: 3-hydroxypiperidin-2-one and 3-methylthiazolidine-4-carboxylic acid.

**Figure 2:**
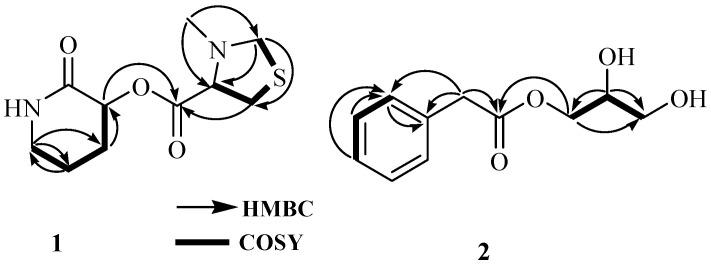
COSY and HMBC correlations for **1** and **2**.

The presence of 3-hydroxypiperidin-2-one was confirmed by the analysis of 1D and 2D NMR spectra. Analysis of the COSY spectrum revealed one coupling sequence from H-2 to H_2_-5 (Figure 2 and Supporting Information); ^13^C-NMR revealed five carbon resonances, including one amide peak (*δ*_c_ 172.8, C-1) and one NH proton resonance (*δ*_H_ 8.23, brs) in the ^1^H NMR spectrum measured in DMSO-*d*_6_ (Table 1 and Supporting Information). The NH proton is an exchangeable proton; so its resonance did not appear in the ^1^H NMR spectrum recorded in CD_3_OD but was observed in DMSO-*d*_6_ (Supporting Information); all these resonances were attributed to 3-methylthiazolidine-4-carboxylic acid.

Similarly, the presence of 3-methylthiazolidine-4-carboxylic acid also was confirmed by the analysis of 1D and 2D NMR spectra. Analysis of the COSY spectrum revealed one coupling sequence from H-2′ to H_2_-5′ where H_2_-5’ ((Figure 2 and Supporting Information) (*δ*_H_ 2.93, m) showed *W*-coupling with H_2_-3′ (*δ*_H_ 2.26, m). The position of the methyl group (*δ*_c_ 38.3, C-7′; *δ*_H_ 2.67, s, H_3_-7′) was determined by the HMBC correlation (Figure 2). In the case of the 5-membered pyrrolidine ring, the sulfur atom is always present as a thioproline ring system in a natural product [21]. ^13^C-NMR revealed five carbon resonances, including one carbonyl carbon peak (*δ*_c_ 167.3, C-1′) and N-CH_3_ carbon resonance (*δ*_c_ 38.3, C-7′). ^1^H NMR revealed one methyl (*δ*_H_ 2.67, s, H_3_-7′), two methylene (*δ*_H_ 2.26, m; *δ*_H_ 2.93, m), and methine (*δ*_H_ 4.36, t, *J* = 4.5) proton resonances; all these resonances were attributed to 3-methylthiazolidine-4-carboxylic acid.

**Figure 3:**
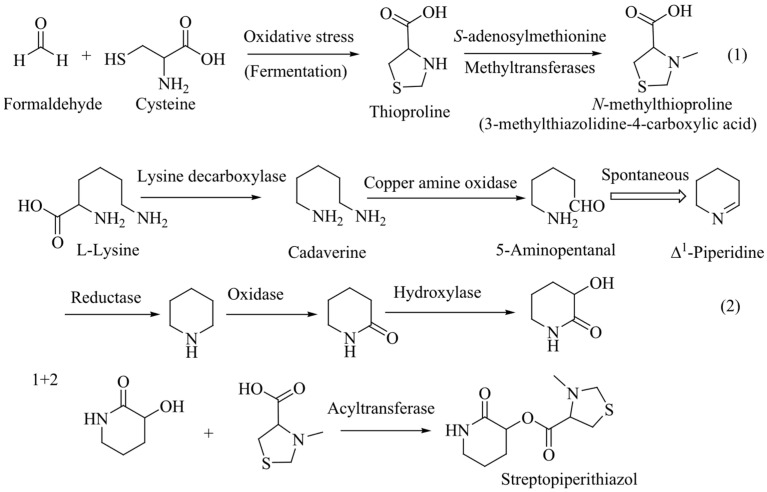
Probable biosynthetic pathway of streptopiperithiazol.

Finally, 3-hydroxypiperidin-2-one and 3-methylthiazolidine-4-carboxylic acid units were joined by ester linkage, which was confirmed by HBMC correlation from H-2 to C-1′. Four degrees of unsaturation were attributed to two carbonyl carbons (one for each) and two ring systems (one for each) required by the molecular formula of 1. ROESY correlation was observed between H-2 and H-2’, indicating that H-2 and H-2′ were located on same side. Compound **1**, named streptopiperithiazol, has an unusual carbon skeleton among the thioproline moiety-containing natural products. Due to lack of proper facilities and limited yield, absolute configuration of 1 was not established. A probable biosynthetic pathway of 1 is given in Figure 3.

The molecular formula of 2 was determined to be C_11_H_14_O_4_ by the positive HRESIMS ion at *m*/*z* 233.0786 [M + Na]^+^ (Supporting Information). The UV absorption bands at 202 and 257 nm indicated the presence of carbonyl and benzene chromophores, respectively. The IR spectrum suggested the presence of hydroxy (3390 cm^−1^) and carbonyl (1726 cm^−1^) groups in the molecule. The gross structure of 2 was determined based on 1D and 2D spectroscopic data analysis (Figure 2). In the ^1^H NMR spectrum, the resonances for one methylene proton (*δ*_H_ 3.68, s), monosubstituted aromatic protons between *δ*_H_ 7.24 and 7.30, and a pattern of signals between *δ*_H_ 3.52 and 4.16, which is the characteristic of a monosubstituted glycerol moiety, were observed (Table 1). Analysis of the COSY spectrum exhibited two spin systems: one from H-4 to H-6 and another from H_2_-1′ to H_2_-3′. An HMBC correlation of H_2_-1′ with a carbonyl carbon C-1 (*δ*_c_ 173.5) revealed the ester linkage between 2-phenylacetic acid and the glycerol moiety. The absolute configuration of the stereogenic center at C-2′ was determined to be *S* based on comparing the optical rotation value ([α]^23^_D_–16) with that of an identical compound, (2′*S*)-hydroxypropan-2′,3′-diol orsellinate ([α]^20^_D_–17) [9]. The structure of compound **2** was elucidated as (2′*S*)-2′,3′-dihydroxypropyl 2-phenylacetate and named streptobenzoglyceride. 

Compounds **1** and **2** were subjected to an antimicrobial test against two Gram-positive bacteria (*Bacillus subtilis* and *Staphylococcus aureus*), two Gram-negative bacteria (*Escherichia coli* and *Pseudomonas aeruginosa*), and one yeast (*Saccharomyces cerevisiae*). Minimum inhibitory activity (MIC) was shown against these pathogens in the range of 16–128 μg/mL, where the highest activity was shown against *B. subtilis* and *S. cerevisiae* at 16 μg/mL (Table 2).

Marine bacteria produce a diversified chemical structure containing small, antimicrobial molecules, including antibiotics that can inhibit or kill both Gram-positive and Gram-negative bacteria, as well as fungi and other microorganisms. These molecules are mainly metabolic products of marine bacteria, particularly within the Actinobacteria phylum, and are being explored for their potential in combating infectious diseases and antibiotic resistance [22]. 

Tirandamycins A and B were isolated from the crude extract of *Streptomyces tirandamycinicus* sp. and showed potent antibacterial activity against *Streptococcus agalactiae* with MIC values of 2.52 and 2.55 μg/mL, respectively [23]. Mersaquinone, a new derivative of tetracene, was isolated from *Streptomyces* sp. obtained from a marine sediment sample and showed MIC at a concentration 3.36 μg/mL against an MRSA strain [24]. Collismycin C isolated from the fermentation supernatant of a marine-derived *Streptomyces* sp. MC025 exhibited potent antibiofilm activity against both MSSA and MRSA at 50 μg/mL.

Most of the naturally occurring diketopiperazines and peptides contained a proline moiety is remained in a cyclic form rather than an open form [25,26]. Proline derivatives exhibit a wide range of antimicrobial activities [12,13]. Prolines derivatized with *N*-methylthioprolines are rarely found in natural sources. Compound **1** has an unusual carbon skeleton among the proline moiety-containing natural products, which showed meaningful activity against tested pathogenic bacteria (Table 2). As this type of compound from both natural and synthetic sources is rarely found, the scope for comparing antimicrobial activities with literature values is limited.

Monoglycerides (MGs) showed antimicrobial activities against a wide range of pathogenic bacteria, enveloped viruses, and yeast [27]. MGs exhibit more antimicrobial activity than free fatty acids with a medium-length side chain (C_8_–C_12_) [28,29]. Gram-positive bacteria are more sensitive to MGs than Gram-negative bacteria [30]. MGs are lipophilic compounds and therefore their primary site of attacking is the cytoplasmic membrane of microbial cells. As the cytoplasmic membrane of Gram-positive bacteria contains more lipids compared to Gram-negative bacteria, Gram-positive bacteria are probably more affected by MGs than Gram-negative bacteria. Although the precise mechanism of MGs for antibacterial activity has not been fully understood, it is assumed that in Gram-positive bacteria, monoglycerides bind with the cytoplasmic membrane and thereby inhibit the transport of amino acids, which leads to damage of the bacterial cells [31,32]. Bacterial cell damage has also been shown when grown in the presence of MGs [33,34]. The activity spectrum of MGs can also be broadened against Gram-negative bacteria in combination with EDTA or citrate. It has been shown that MGs suppress the antibiotic resistance gene and thereby slow the development of resistance in bacteria [23,24].

Due to the non-toxic property in low concentrations, MGs may be used as additives or alternatives to antibiotics for treatment of infectious diseases [35,36,37]. The interesting point to be noted is that although compound **2** is a monoglycerol ester, it is significantly active against both Gram-positive and Gram-negative pathogenic bacteria (Table 2) compared to the literature values (monocaprylin, 11 mg/mL) [36]. Compound **2** was synthesized through lipase-mediated desymmetrization of glycerol with aromatic anhydrides, and its ^1^H and ^13^C resonances are so ambiguous [38]. Furthermore, the antimicrobial activity of 2 has not been reported yet. Compound **2** was isolated for the first time from natural sources. For this reason, the detailed characterization and antimicrobial activity of **2** have been presented here. 

## 3. Materials and Methods

### 3.1. General Experimental Procedures

Solvents used in HPLC were distilled prior to use. Flash chromatography was carried out on C18 reversed-phase gel (100–200 mesh). Optical rotations were measured on a JASCO (DIP-1000) digital polarimeter (JASCO Corp., Tokyo, Japan) at the sodium lamp (λ = 589 nm) D line. UV spectra were obtained on a Shimadzu UV-1650PC spectrophotometer (Shimadzu Corporation, Tokyo, Japan). IR spectra were recorded on a JASCO FT/IR-4100 spectrophotometer ((JASCO Corp., Tokyo, Japan). ^1^H and ^13^C NMR were recorded on a Varian Unity 500 spectrometer at 500 and 125 MHz, respectively. Chemical shifts (*δ*) are reported in parts per million (ppm) referenced to CH_3_OH at 3.30 ppm for ^1^H and CD_3_OD at 49.0 ppm for ^13^C and to (CH_3_)_2_SO at 2.50 ppm. Coupling constants are reported in Hertz (Hz), with the following abbreviations used: s = singlet, brs = broad singlet, dd = double doublet, t = triplet, m = multiplet. One-dimensional and two-dimensional NMR spectra were acquired on a Varian Unity 500 spectrometer (Agilent Technologies, Santa Clara, CA, USA). High-resolution mass spectra were recorded on a hybrid ion-trap time-of-flight mass spectrometer (Shimadzu LC/MS-IT-TOF) (Shimadzu Corporation, Tokyo, Japan). Melting point was determined on a Fisher-Johns melting point apparatus. Analytical HPLC was conducted with a PrimeLine binary pump with a RI-101 (Shodex) (Resonac Corporation, Tokyo, Japan) and variable UV detector (M 525) (Resonac Corporation, Tokyo, Japan). Continuous centrifugation was conducted on a Centrifugal Separator, Kansai Centrifugal Separator Manufacturing Co., Ltd. (Osaka, Japan). Semi-preparative HPLC was performed using ODS (YMC-Pack-ODS-A, 250 × 10 mm i.d, 5 µm) and silica (YMC-Pack-SIL, 250 × 10 mm i.d, 5 µm) columns. Analytical HPLC was conducted on an ODS column (YMC-Pack-ODS-A, 250 × 4.6 mm i.d, 5 µm). Natural sea water (NSW) was collected from the East China Sea of South Korea.

### 3.2. Isolation and Taxonomy of the Strains 04DH31 and 06CH80

A sediment sample was collected from Chuuk Lagoon atoll system south of Uman Island, the Federated States of Micronesia. The sample site was located near the shipwreck of the WW II freighter, the *Sankisan Maru*, about 500 m off the southern end of Uman Island at a depth of 30 m (7°00′–7°21′9 N, 151°85′ E). Sediment sample material was collected by scuba diving. The pH and salinity of the sample collection site were 7.8 and 32 ppt, respectively. The sample was kept in sterile plastic bag and kept in 4 °C before use. Strains 04DH31 and 06CH80 were isolated from the sediment sample by a serial dilution technique. In brief, one gram of the sediment sample was diluted in sterilized sea water (10^−1^, 10^−2^, 10^−3^ and 10^−4^) in aseptic conditions, and 100 µL from each dilution was spread on modified Bennett’s agar medium (0.1% yeast extract, 0.1% beef extract, 0.2% tryptone, 1% dextrose, 100% natural sea water, 1.8% agar, and pH adjusted to 7.2 before sterilization). The plates were incubated at 30 °C, and the resulting colony of strains was isolated and maintained on the modified Bennett’s agar. The strains 04DH31 (GenBank Accession No. KJ371986) and 06CH80 (GenBank Accession No. KJ371985) were identified as *Streptomyces* spp. based on their 16S rDNA sequence analysis.

### 3.3. Seed and Large-Scale Cultures of the Strains 04DH31 and 06CH80

The seed and mass cultures of the strains 04DH31 and 06CH80 were carried out in modified Bennett’s broth medium. The compositions and pH of the seed culture medium (0.1% yeast extract, 0.1% beef extract, 0.2% tryptone, 1% dextrose, salinity 20 g/L, pH 7.6) were the same as those of the mass culture medium. Then, 200 mL of the medium was dispensed in 500 mL conical flask and sterilized. A single colony of strains 04DH31 and 06CH80 from the agar plates was inoculated aseptically into two separate flasks and incubated at 28 °C for 2 days on a rotary shaker at 120 rpm. An aliquot (0.2% *v*/*v*) from the seed culture was inoculated aseptically into 2 L flasks (40 flasks for each strain) containing 1 L sterilized culture medium. The production culture (40 L each) was incubated in the same conditions as the seed culture for 7 days and then harvested. 

### 3.4. Extraction and Isolation

The production culture broth of the strain 04DH31 was centrifuged, and the supernatant was extracted with EtOAc (2 × 40 L). The EtOAc layer was concentrated to dryness using rotary evaporators at 40 °C. The residual suspension (2.1 g) was subjected to ODS open column chromatography followed by stepwise gradient elution with MeOH–H_2_O (*v*/*v*) (1:4, 2:3, 3:2, 4:1 and 100:0) as the eluent. The fraction eluted with MeOH–H_2_O (2:3 *v*/*v*) was again divided into ten fractions (F-1–10) by an ODS MPLC (medium-pressure liquid chromatography) using 0–40% MeOH in H_2_O as the eluent (the retention time of F-9 was 45 min). Compound **1** (2 mg) was isolated from F-9 by a semi-preparative ODS HPLC using 10% MeOH in H_2_O as the eluent (retention time 20 min). Compound **2** was isolated from EtOAc (2.6 g) of the culture broth of the strain 06CH80 in a similar way. The fraction eluted with MeOH–H_2_O (2:3 *v*/*v*) was again subjected to the ODS MPLC (10–50% MeOH in H_2_O) to obtain nine fractions ((F-1–9). By repeated HPLC steps, compound 2 was obtained in pure form (1.7 mg) from the fraction F-7.

**Streptopiperithiazol (1):** White crystal; [α]^23^_D_–58 (*c* 0.05, MeOH); mp 130 °C; UV (MeOH) λ_max_ (log ε) 202 (4.46) and 278 (3.15) nm; IR (MeOH) *ν*_max_ 2861, 1652, 1054 cm^−1^; ^1^H and ^13^C NMR data (CD_3_OD), see Table 1; HRESIMS *m/z* 267.0777 [M + Na]^+^ (calcd for C_10_H_16_N_2_O_3_SNa, 267.0779).

**Streptobenzoglyceride (2):** Amorphous solid; [α]^23^_D_–16 (*c* 0.05, MeOH); UV (MeOH) λ_max_ (log ε) 203 (4.27), 252 (3.03), 257 (2.9) and 263 (3.10) nm; IR (MeOH) *ν*_max_ 3390, 2931, 1726 cm^−1^; ^1^H and ^13^C NMR data (CD_3_OD), see Table 1; HRESIMS *m/z* 233.0786 [M + Na]^+^ (calcd for C_11_H_14_O_4_Na, 233.0790).

### 3.5. Molecular Identification of 04DH31 and 06CH80 Strains

#### 3.5.1. Isolation and Purification of Genomic DNA

04DH31 and 06CH80 strains were grown in Bennett’s agar medium at 30 °C for 4 days. An isolated colony of 04DH31 and 06CH80 was inoculated into 30 mL Bennett’s broth medium and incubated at 30 °C at 150 rpm for 2 days. The cells were harvested by centrifugation (10 mm, 5000 rpm), and the cell pellets were resuspended in 5 mL lysis buffer solution (75 mM NaCl, 25 mM EDTA, 20 mM Tris-HCl buffer, pH 7.5). Then lysozyme was added at a concentration of 1 mg/mL and incubated at 37 °C for 30 min. Then 500 μL of 10% SDS (sodium dodecyl sulfate) and 0.5 mg/mL proteinase K (Promega Korea, Seoul, Republic of Korea) were added and incubated at 55 °C with occasional inversion for 2 h. Then 2 mL of 5M NaCl solution and 5 mL chloroform were added and incubated at room temperature for 30 min with frequent inversion. Then the sample was centrifuged at 5000 rpm for 15 min, and the aqueous phase was transferred to a new tube using a blunt-ended pipette tip. DNA was precipitated by adding 5 mL isopropanol, and the tube was gently inverted. Then DNA was transferred into a microcentrifuge tube, rinsed with 70% ethanol, dried in vacuum, and dissolved in 100 μL TE buffer (Tris-EDTA) (10 mM Tris-HCl, 1 mM EDTA, pH 8.0).

#### 3.5.2. 16S rDNA Sequencing

The 16S rDNA gene was then amplified by the following pairs of primers: forwards: 5′-GAGTTTGATCCTGGCCAG-3′ and reverse: 5′-AAGGAGGTGATCCAGCC-3′ using a PCR kit (Sino-American Biotechnology, San Diego, CA, USA) [39]. The PCR reaction mixture contained 2 μL from each primer and 10 µL template DNA and adjusted to a final volume of 50 µL by distilled water. The DNA amplification was performed through the following program: denaturation at 95 °C for 5 min followed by 35 cycles for 1 min, annealing at 56 °C for 1 min, and extension at 72 °C for 3 min. At the end of the cycles, the reaction mixture was kept at 72 °C for 5 min and then cooled to 4 °C. The amplified 16S rDNA fragment was separated by agarose gel electrophoresis. Electrophoresis was carried out on 1% agarose gel containing ethidium bromide (0.5 μg/mL) and detected by a Gel documentation system. The purified fragment was directly sequenced by using a *Taq* DyeDeoxy terminator cycle sequencing kit (Applied Biosystems, Foster City, CA, USA). The following sequencing primers were used: 5′-TAAGGAGGTGATCCAGCC-3′, 5′-TGCTGGCAACACAG AACAAG-3′, and 5′-ACTCTG CCTGCCCGTATCG-3′.

#### 3.5.3. Sequence Alignment and Phylogenetic Tree Construction

Multiple sequence alignment and phylogenetic analysis were performed using MEGA 11 tools to evaluate the sequences and determine their evolutionary connections [40]. The UPGMA methods were utilized during the analysis [41]. Nukleotide sequences were compared with those available in the GenBank database through NCBI Blast. For phylogenetic analysis, sequences were aligned with those of the reference strains. The phylogenetic tree was derived from distance matrices using the neighbor-joining method (see Supplementary Materials, Figures S1–S14).

### 3.6. Antimicrobial Assays

The minimum inhibitory concentrations of compounds **1** and **2** were determined by using a conventional broth dilution assay. Antibacterial and antiyeast tests were performed in nutrient broth (the medium composition was 0.3% beef extract, 0.5% peptone, and pH 7.2 before sterilization) and yeast maltose broth (1% dextrose, 0.3% beef extract, 0.5% peptone, 0.3% malt extract, and pH 7.2 before sterilization), respectively. An overnight broth culture of each pathogenic strain was prepared, and the final concentration of microorganisms in each culture was adjusted to 1.5 × 10^8^ cfu/mL by comparing the culture turbidity with the 0.5 McFarland standard. A serial two-fold dilution of each compound was prepared in 96-well microtiter plates over the range of 0.5–256 μg/mL. At first, 100 μL of respective culture medium was placed in each well, and 100 μL of sample (concentration of 1024 μg/mL) was added to the first well and mixed. Then 100 μL was transferred from the first well to the second well and mixed. In this way, the sample was diluted until the final concentration in the range of 0.5–256 μg/mL was obtained after addition of 70 μL of medium and 30 μL of culture broth. Broth media without the compound served as a control. The plates were incubated for 24 h at 37 °C for bacteria and 48 h at 30 °C for the yeast. Changes in turbidity were correlated with changes in cell numbers. The lowest concentration of antimicrobial agent at which no visible turbidity was observed was taken as the minimum inhibitory concentration.

## 4. Conclusions

In conclusion, two marine sediment-derived *Streptomyces* spp., which were morphologically interesting and showed good antimicrobial activity at the preliminary screening, were cultured in modified Bennett’s broth medium with a view to isolating the bioactive components. The EtOAc extracts obtained from the culture broths were subjected to repeated chromatographic steps leading to the isolation of compounds **1** and **2**. The structures of **1** and **2** were elucidated based on spectroscopic data. Both compounds significantly inhibited the tested pathogens compared to the literature values. Optimization of the antimicrobial activity through the synthesis of derivatives of these compounds may lead to the development of new antimicrobial drugs.

## Figures and Tables

**Table 1:** ^1^H and ^13^C NMR data of compounds **1** and **2** in CD_3_OD.

No.	1		2	
	*δ*_c_	*δ*_H_, mult. (*J* in Hz)	*δ*_c_	*δ*_H_, mult. (*J* in Hz)
1	172.8		173.5	
2	60.5	4.26, t (7.0)	41.8	3.68, s
3	29.4	2.03, m 2.33, m	135.6	
4/8	23.6	1.97, m 2.33, m	130.4	7.30, m
5/7	46.5	3.53, m	129.5	7.27, m
6		8.23, brs ^a^	128.0	7.24, m
1′	167.3		66.9	4.08, dd (11.3, 6.0) 4.16, dd (11.3, 4.5)
2′	55.3	4.36, t (4.5)	71.0	3.81, m
3′	24.0	2.26, m	64.0	3.52, dd (5.7, 2.0)
5′	50.1	2.93, m		
6′	38.3	2.67, s		

^a^ Determined in DMSO-*d*_6_.

**Table 2:** Minimum inhibitory concentrations (MICs) of 1 and 2.

Test Organisms	MICs (μg/mL)			
	1	2	AZ	AmpB
*Bacillus subtilis*	16	32	4	-
*Escherichia coli*	64	128	4	-
*Pseudomonas aeruginosa*	32	32	4	-
*Staphylococcus aureus*	16	16	4	-
*Saccharomyces cerevisiae*	128	32	-	2

AZ: Azithromycin (positive control for bacteria); AmpB: Amphotericin B (positive control for yeast); -: not tested.

## Data Availability

The data that support the findings of this study are available from the corresponding author, M.A.M.M., upon reasonable request.

## Supplementary Materials

The following supporting information can be downloaded at: https://www.mdpi.com/article/10.3390/ijms26167771/s1.
