# Supplementary material for: Antimicrobial Unusual Small Molecules from Marine Streptomyces spp
Source: Int J Mol Sci. 2025 Aug 12;26(16):7771. doi: 10.3390/ijms26167771 (PMC12386958; doi:10.3390/ijms26167771)
Supplement: Supplementary file 1 [file ijms-26-07771-s001.zip › ijms-3682688-supplementary.pdf]

Index

**Name of the Figures**	**Page No**
Figure S1: HRESIMS spectrum of compound **1**	03
Figure S2: ^{1}H NMR spectrum of compound 1 in CD3OD	04
Figure S3: ^{1}H NMR spectrum of compound 1 in DMSO-*d*6	05
Figure S4: ^{13}C NMR spectrum of compound **1** in CD3OD	06
Figure S5: COSY spectrum of compound **1** in CD3OD	07
Figure S6: HSQC spectrum of compound **1** in CD3OD	08
Figure S7: HMBC spectrum of compound **1** in CD3OD	09
Figure S8: HRESIMS spectrum of compound **2**	10
Figure S9: ^{1}H NMR spectrum of compound **2** in CD3OD	11
Figure S10: ^{13}C NMR spectrum of compound **2** in CD3OD	12
Figure S11: COSY spectrum of compound **2** in CD3OD	13
Figure S12: HSQC spectrum of compound **2** in CD3O D	14
Figure S13: HMBC spectrum of compound **2** in CD3OD	15
Figure S14: Phylogenic tree of the strains 04DH31 (GenBank Accession No. KJ371986) and 06CH80 (GenBank Accession No. KJ371985)	16

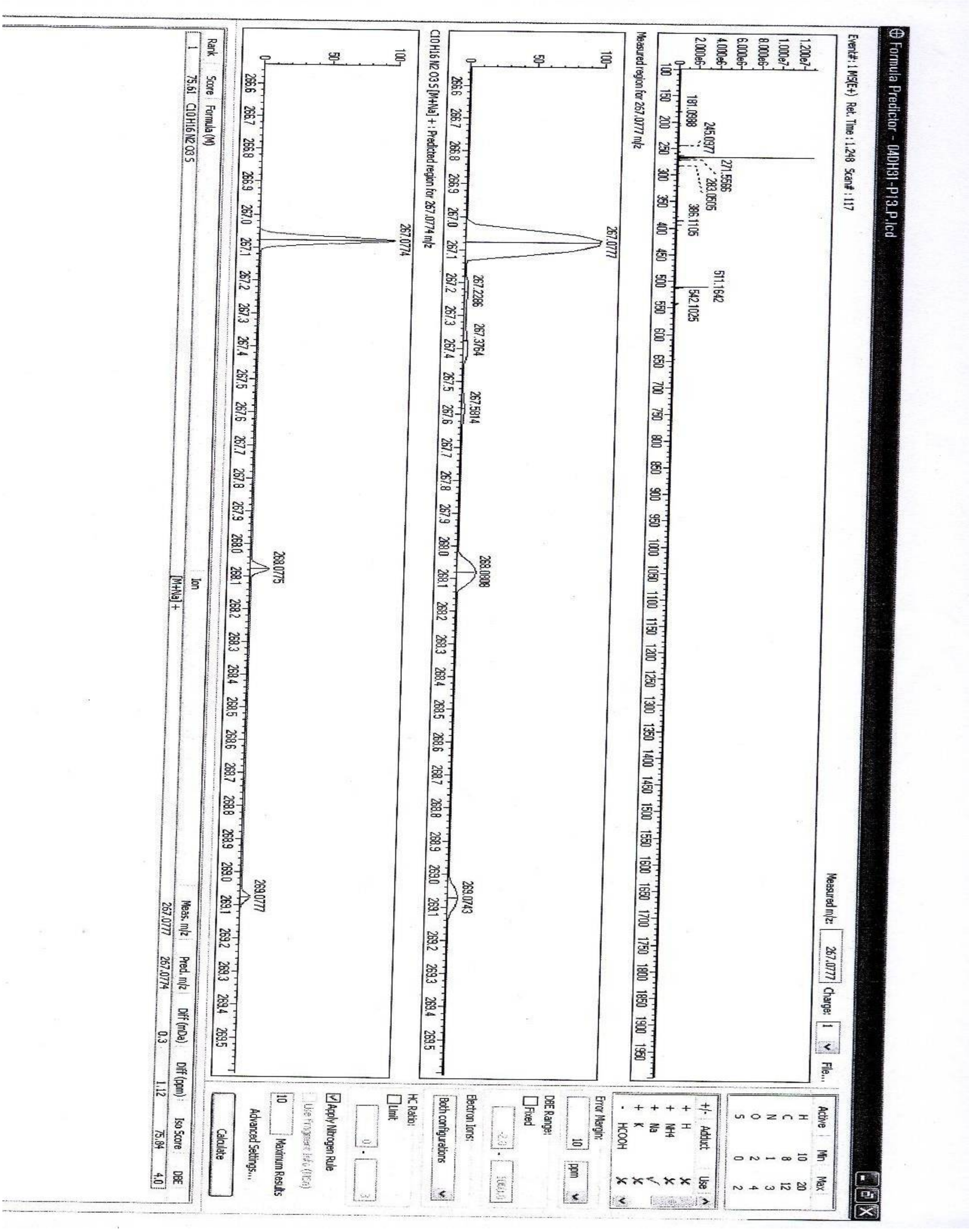

Figure S1. HRESIMS spectrum of compound **1**.

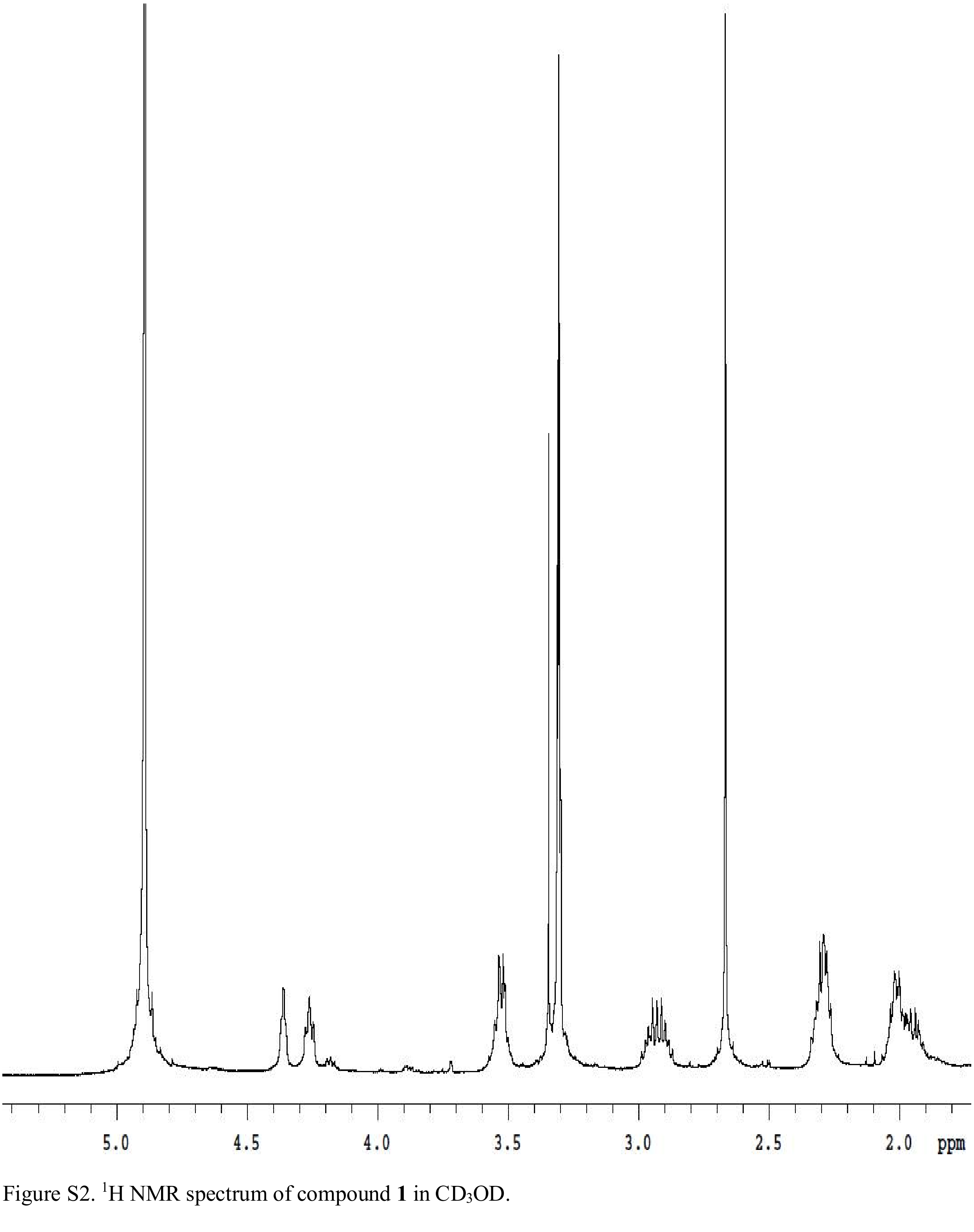

Figure S2. ^{1}H NMR spectrum of compound **1** in CD_3OD.

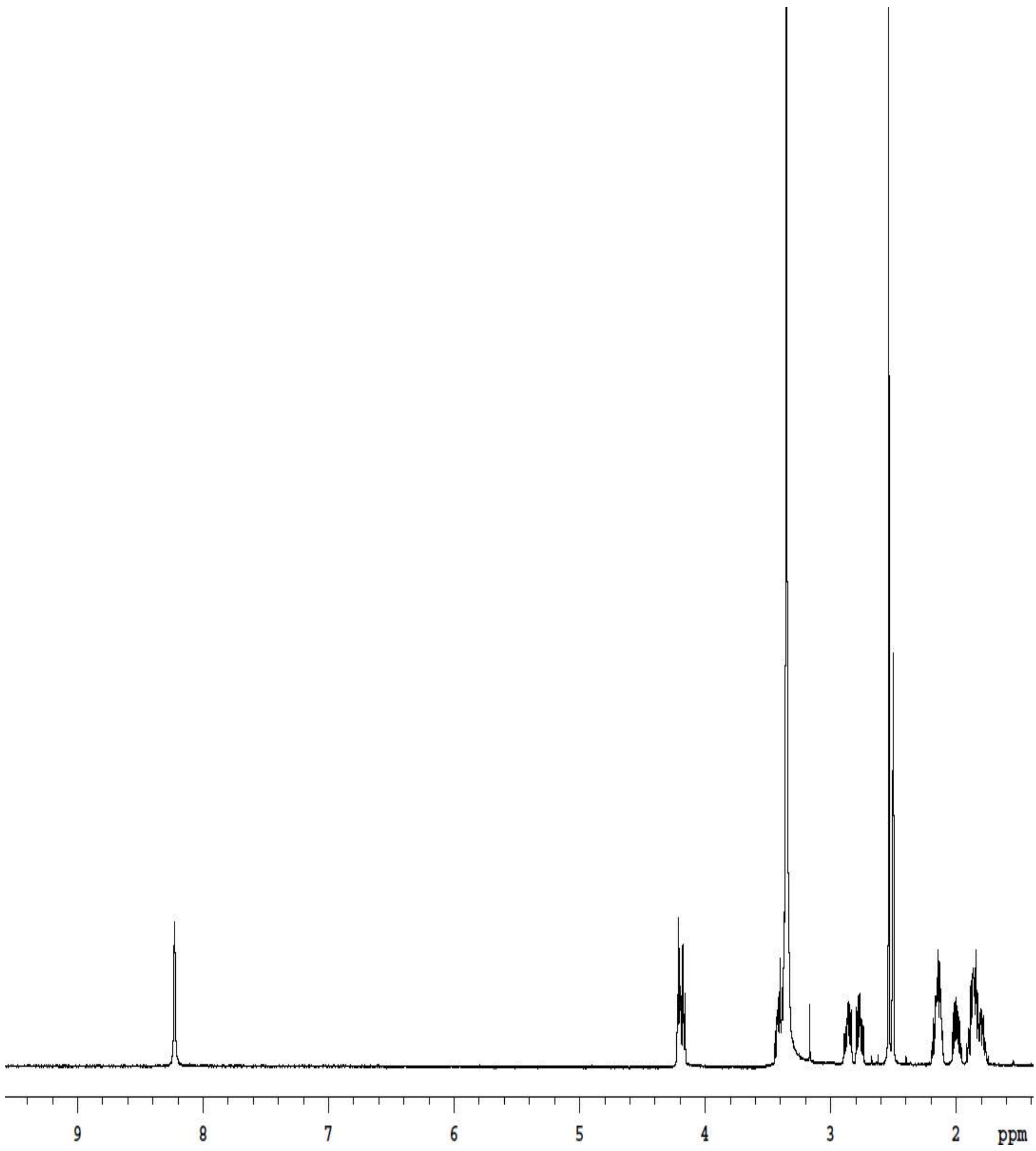

Figure S3. ^{1}H NMR spectrum of compound **1** in DMSO-d_6.

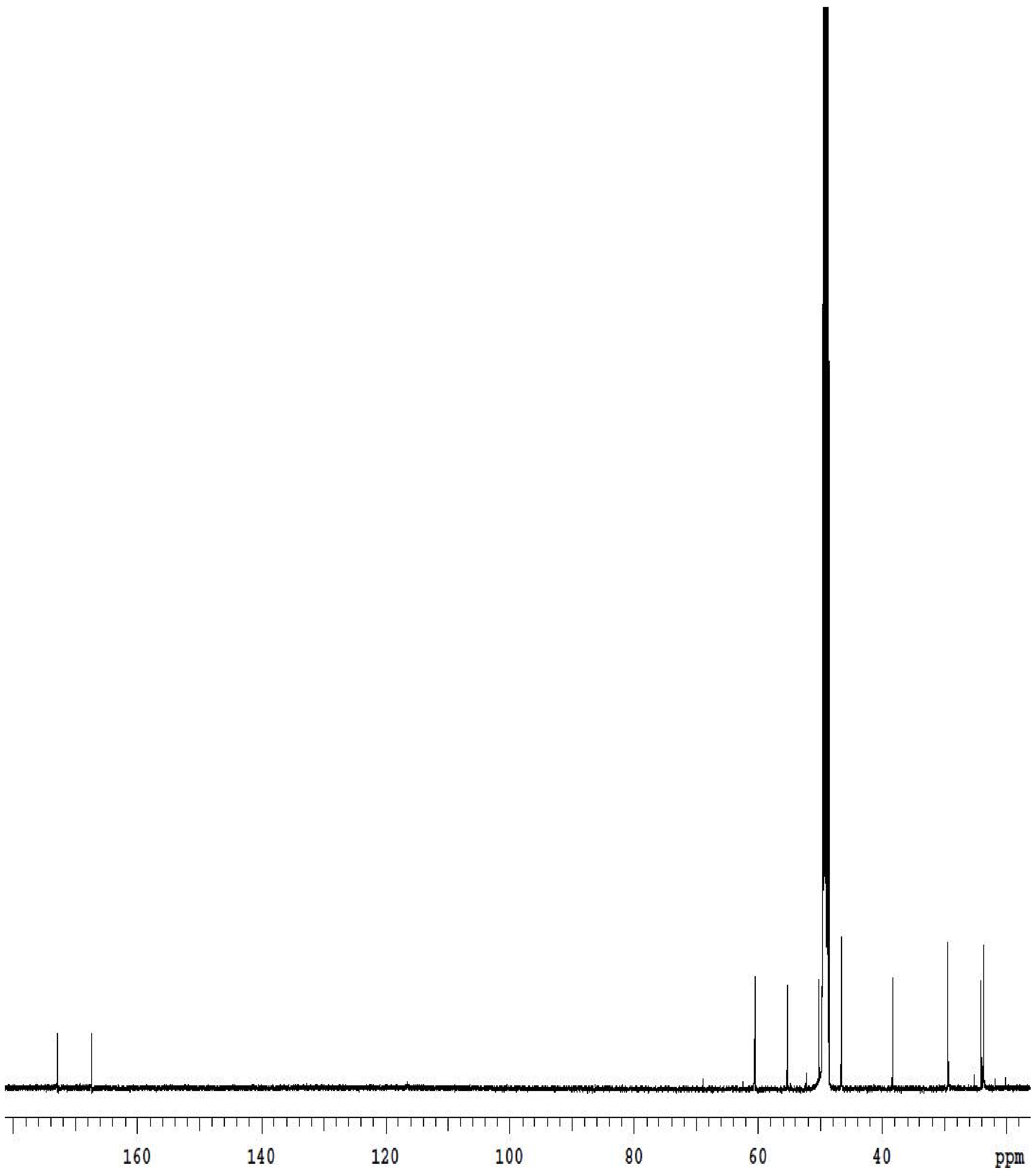

Figure S4. ^{13}C NMR spectrum of compound **1** in CD_3OD.

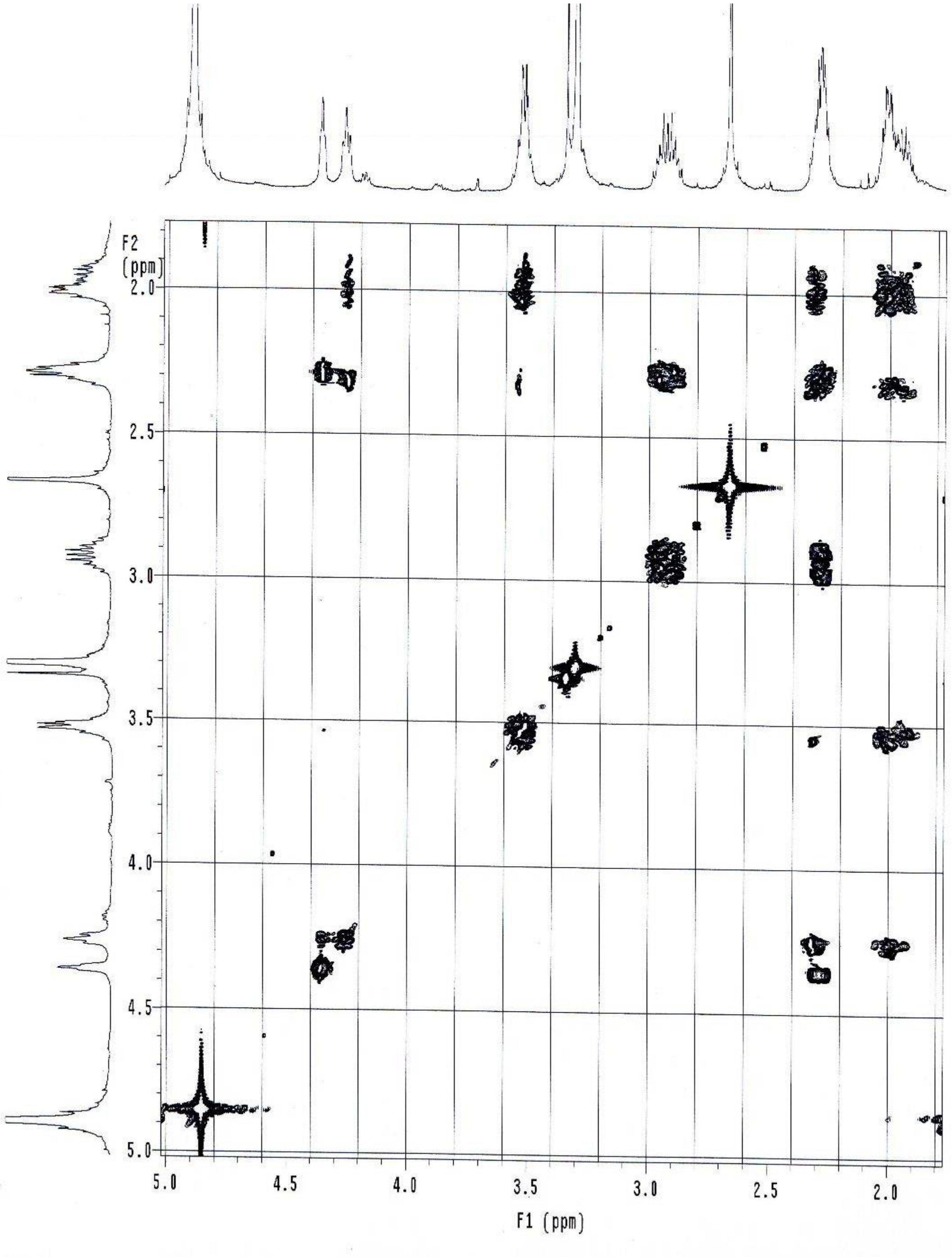

Figure S5. COSY spectrum of compound **1** in CD_3OD.

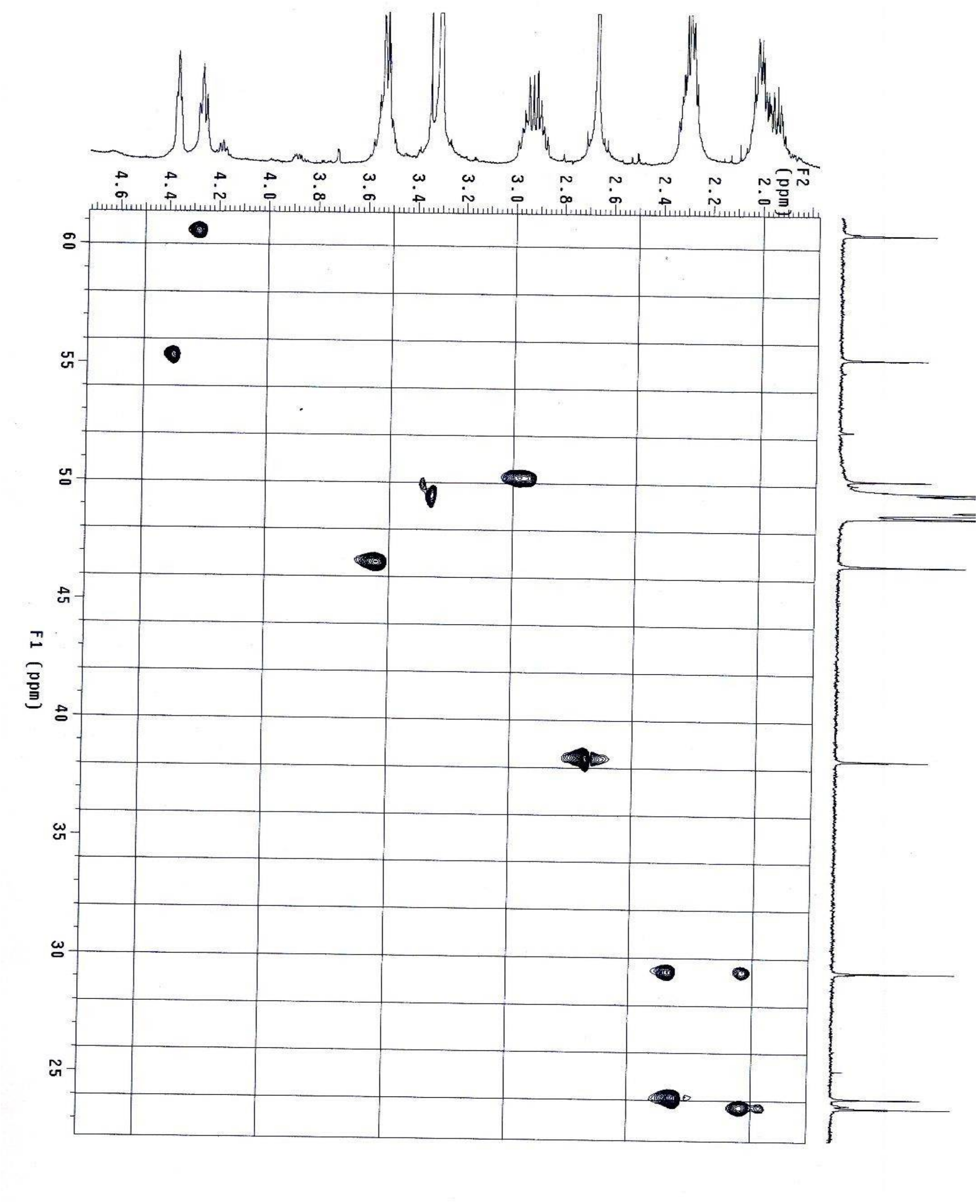

Figure S6. HSQC spectrum of compound **1** in CD_3OD.

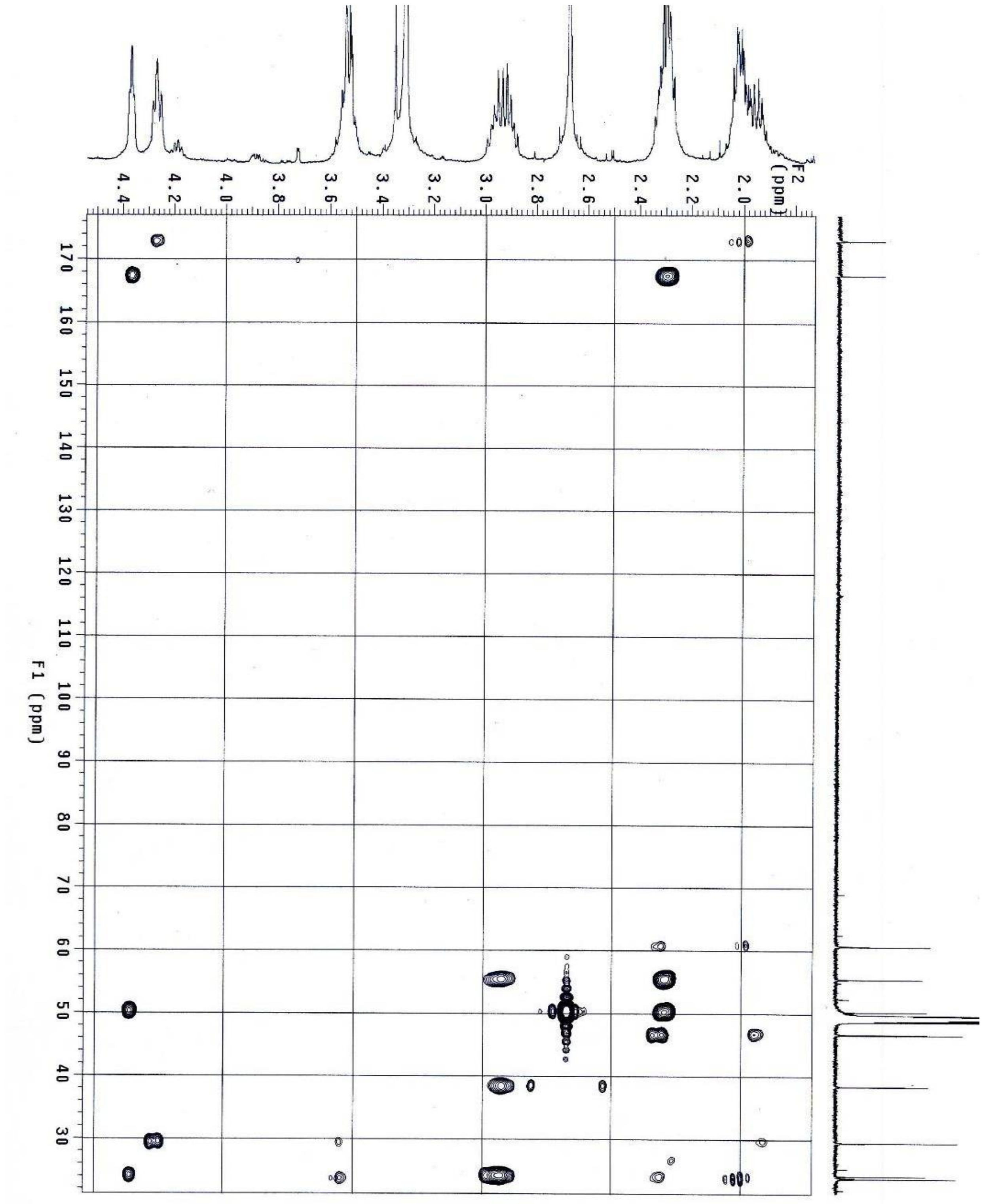

Figure S7. HMBC spectrum of compound **1** in CD_3OD.

Elmt	Val.	Min	Max
H	1	0	300
C	4	0	150
N	3	0	0

Elmt	Val.	Min	Max
O	2	0	12

Use Adduct
Na

Error Margin (ppm):	10	DBE Range:	not fixed	Electron Ions:	both
HC Ratio:	unlimited	Apply N Rule:	yes	Use MSn Info:	no
Max Isotopes:	all	Isotope RI (%):	1.00	Isotope Res:	10000
MSn Iso RI (%):	75.00	MSn Logic Mode:	AND	Max Results:	10

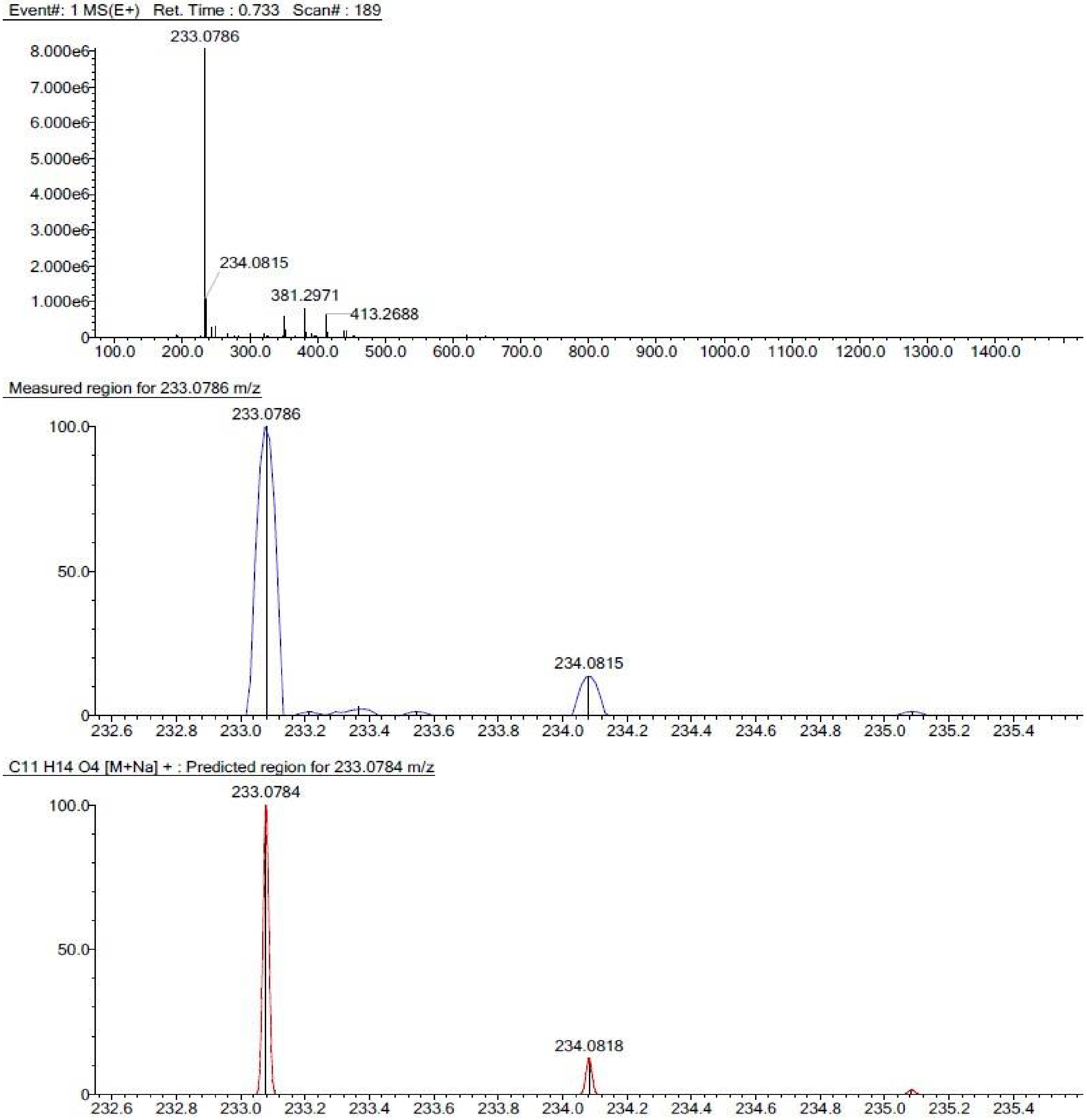

Rank	Score	Formula (M)	Ion	Meas. m/z	Pred. m/z	Df. (mDa)	Df. (ppm)	Iso	DBE
1	71.98	C11 H14 O4	[M+Na] +	233.0786	233.0784	0.2	0.86	71.98	5.0

Figure S8. HRESIMS spectrum of compound **2**.

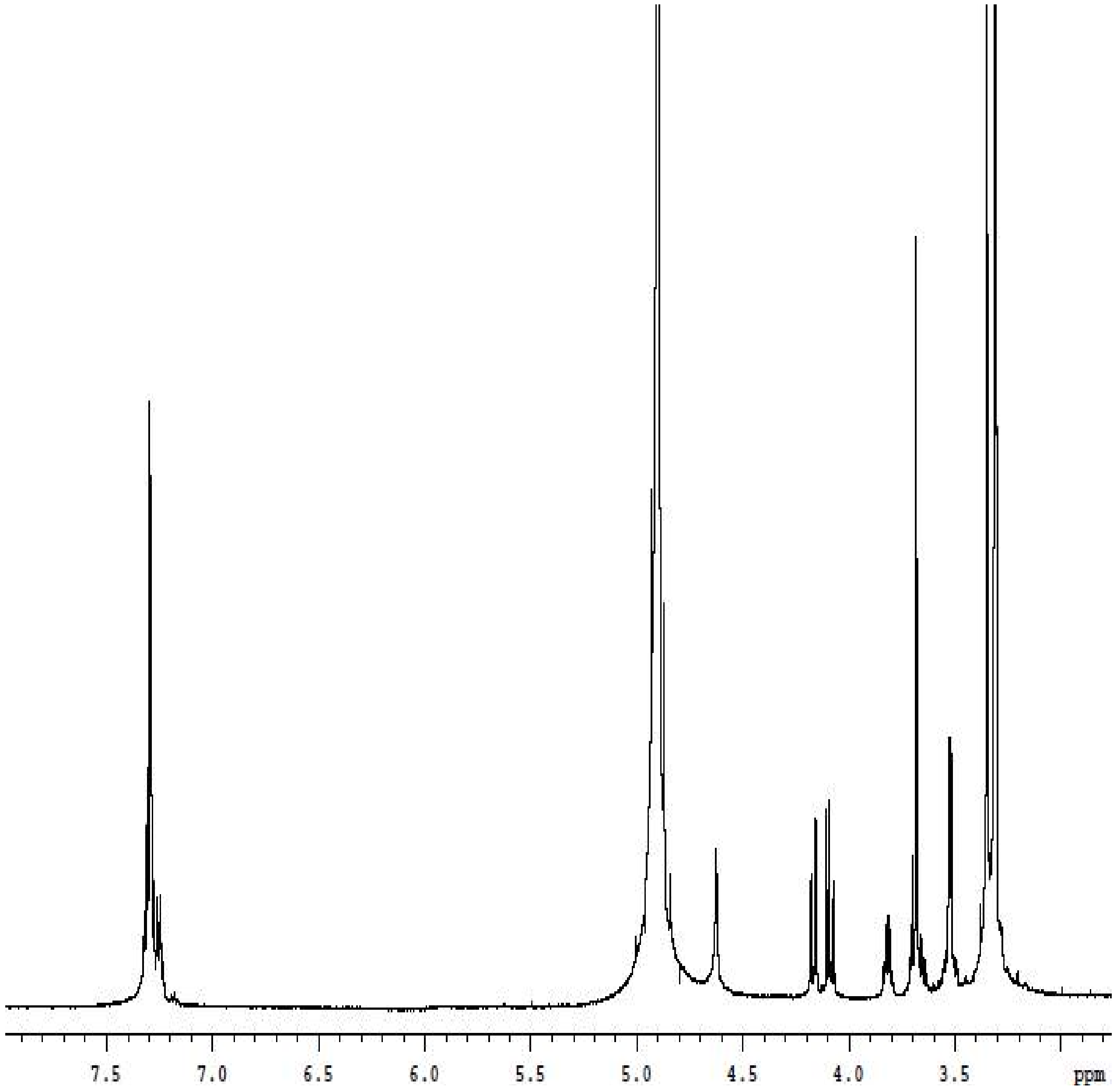

Figure S9. ^{1}H NMR spectrum of compound **2** in CD_3OD.

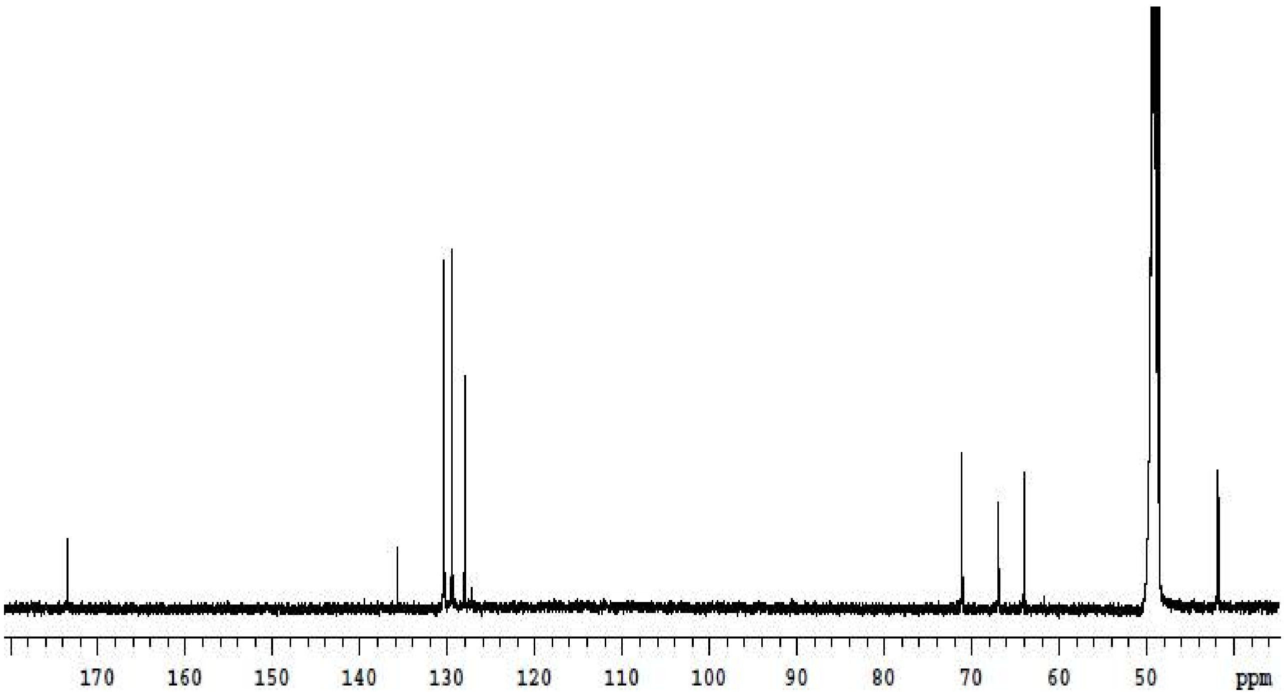

Figure S10. ^{13}C NMR spectrum of compound **2** in CD_3OD.

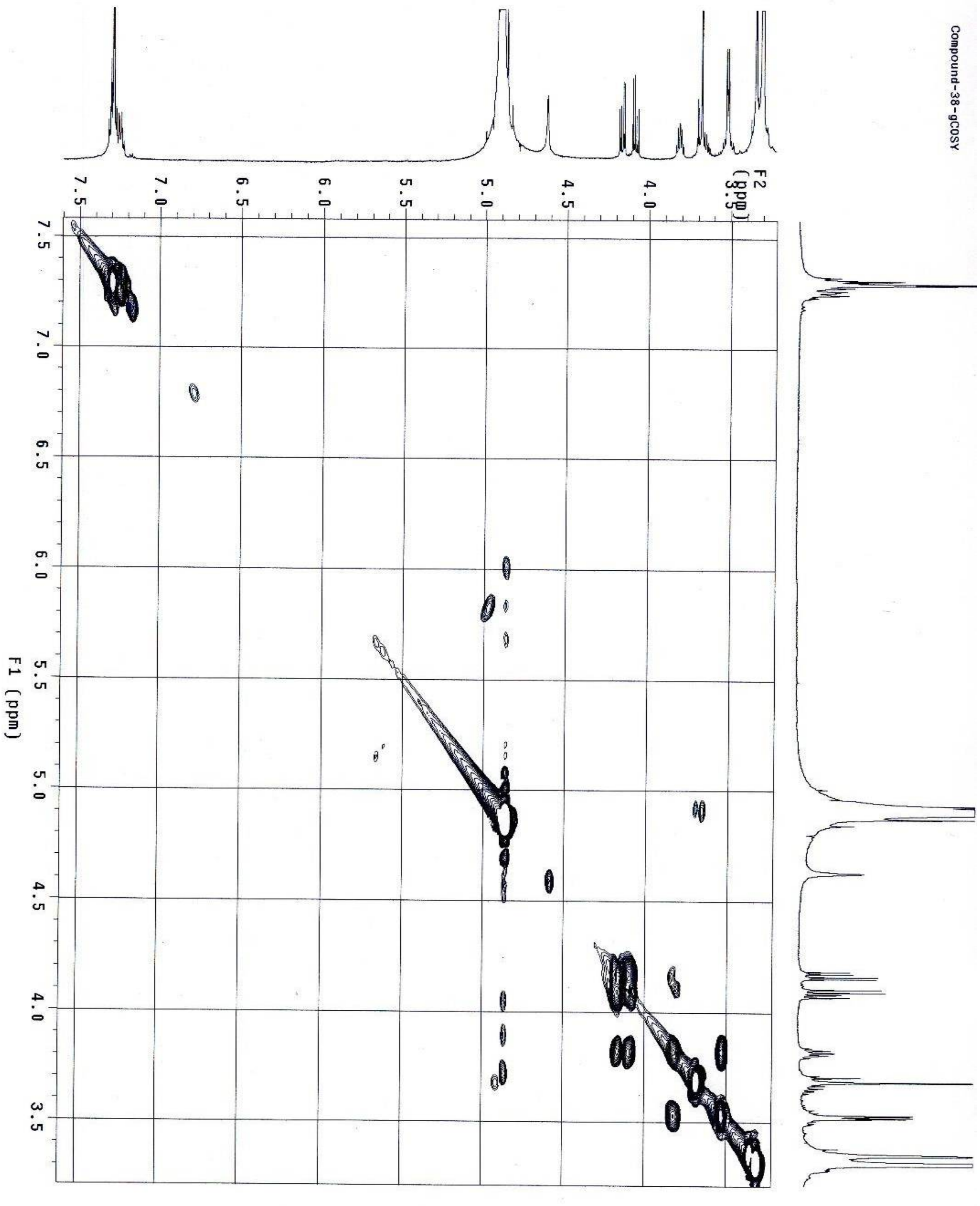

Figure S11. COSY spectrum of compound **2** in CD_3OD.

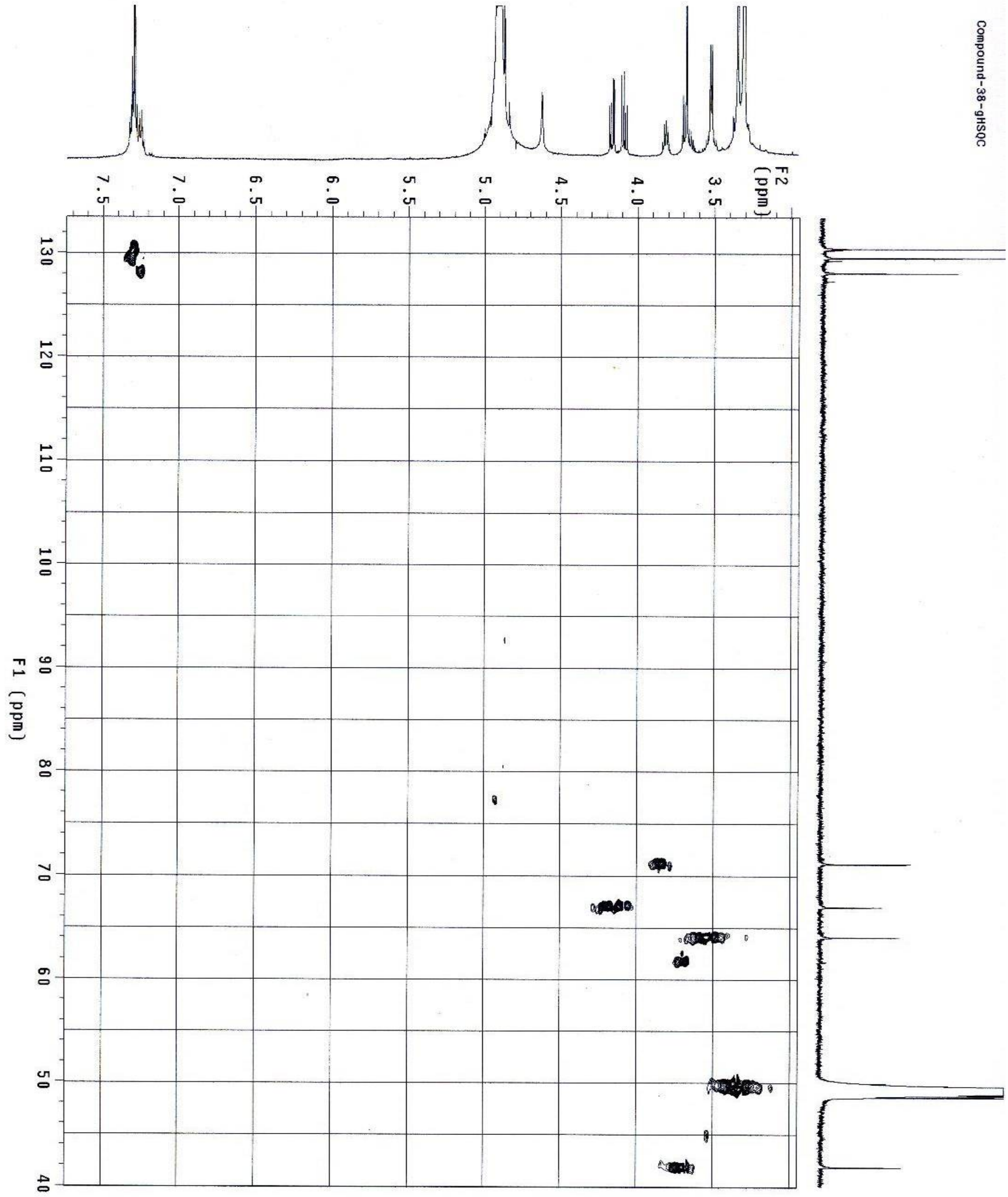

Figure S12. HSQC spectrum of compound **2** in CD_3OD.

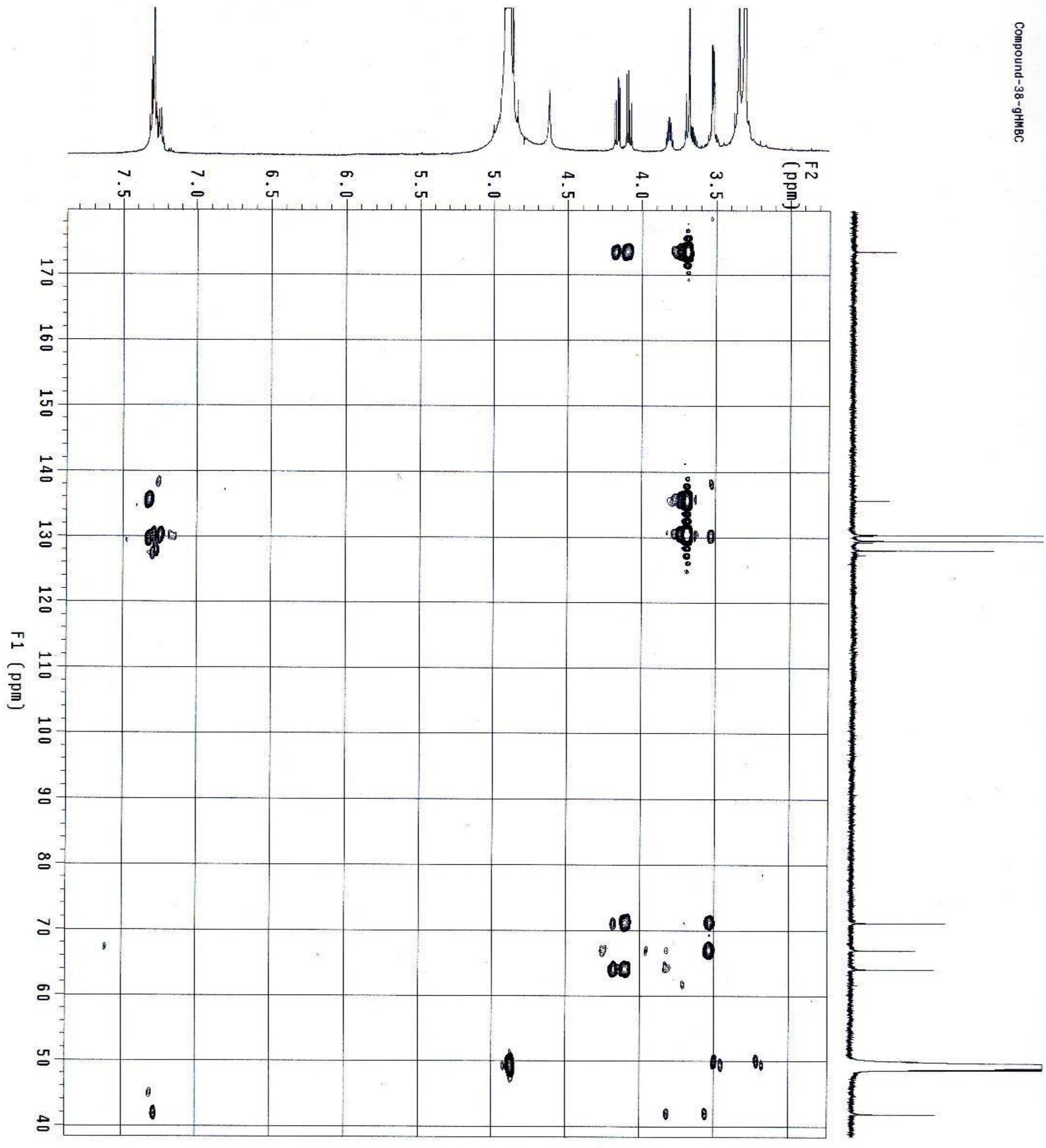

Figure S13. HMBC spectrum of compound **2** in CD_3OD.

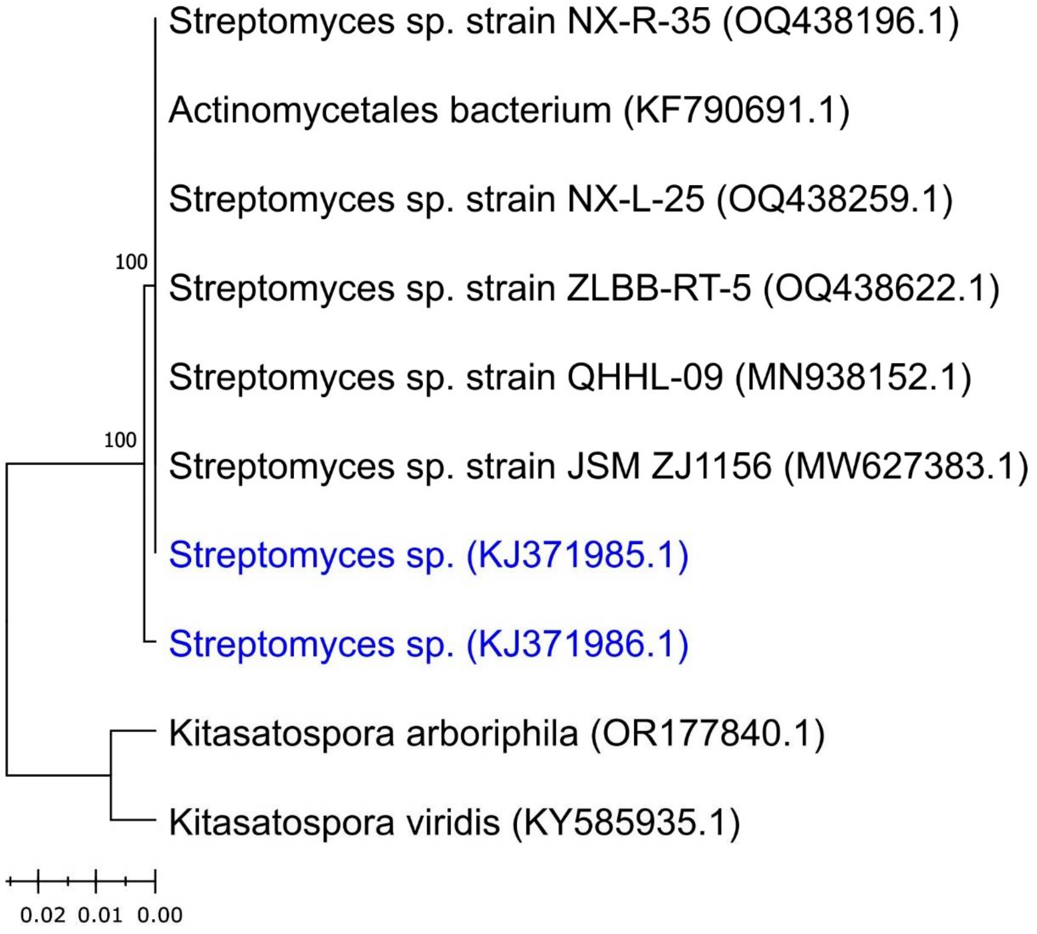

Figure S14: Phylogenic tree of the strains 04DH31 (GenBank Accession No. KJ371986) and 06CH80 (GenBank Accession No. KJ371985)
